# Core elements of serious illness conversations: an integrative systematic review

**DOI:** 10.1136/spcare-2023-004163

**Published:** 2023-06-27

**Authors:** Rebecca Baxter, Susanna Pusa, Sofia Andersson, Erik K Fromme, Joanna Paladino, Anna Sandgren

**Affiliations:** 1 Center for Collaborative Palliative Care, Department of Health and Caring Sciences, Linnaeus University—Vaxjo Campus, Vaxjo, Sweden; 2 Ariadne Labs, Boston, Massachusetts, USA; 3 Harvard Medical School, Boston, Massachusetts, USA; 4 Massachusetts General Hospital, Boston, Massachusetts, USA

**Keywords:** Communication, End of life care, Hospital care, Quality of life, Psychological care, Supportive care

## Abstract

**Background:**

Ariadne Labs’ Serious Illness Care Program (SICP), inclusive of the Serious Illness Conversation Guide (SICG), has been adapted for use in a variety of settings and among diverse population groups. Explicating the core elements of serious illness conversations could support the inclusion or exclusion of certain components in future iterations of the programme and the guide.

**Aim:**

This integrative systematic review aimed to identify and describe core elements of serious illness conversations in relation to the SICP and/or SICG.

**Design:**

Literature published between 1 January 2014 and 20 March 2023 was searched in MEDLINE, PsycINFO, CINAHL and PubMed. All articles were evaluated using the Joanna Briggs Institute Critical Appraisal Guidelines. Data were analysed with thematic synthesis.

**Results:**

A total of 64 articles met the inclusion criteria. Three themes were revealed: (1) serious illness conversations serve different functions that are reflected in how they are conveyed; (2) serious illness conversations endeavour to discover what matters to patients and (3) serious illness conversations seek to align what patients want in their life and care.

**Conclusions:**

Core elements of serious illness conversations included explicating the intention, framing, expectations and directions for the conversation. This encompassed discussing current and possible trajectories with a view towards uncovering matters of importance to the patient as a person. Preferences and priorities could be used to inform future preparation and recommendations. Serious illness conversation elements could be adapted and altered depending on the intended purpose of the conversation.

**WHAT IS ALREADY KNOWN ON THIS TOPIC**

- The Serious Illness Care Program (SICP) and Serious Illness Conversation Guide (SICG) are associated with improved patient outcomes and experiences.
- Serious illness conversation content has been adapted for different patients, clinicians and contexts, yet the core elements of these conversations have not been explored.

**WHAT THIS STUDY ADDS**

- Conversation elements were revealed to be multifaceted with nuanced content that could be altered depending on the intended purpose of the conversation.
- Core conversation elements included having clear intentions and framing, establishing expectations and directions, exploring the current situation and possible trajectory, uncovering matters of importance, elucidating preferences and priorities and supporting preparation and recommendations.
- While modifications have been made to the conversation guide, the same general questions and structure were relevant for most contexts.

**HOW THIS STUDY MIGHT AFFECT RESEARCH, PRACTICE OR POLICY**

- This integrative systematic review contributes important knowledge about core elements of serious illness conversations that can be used in developing or modifying future iterations of the SICP and SICG.
- Informing the core elements for serious illness conversations strengthens the theory supporting the programme and guide and can be used to inform current clinical education and practice.

## Background

 To provide much-needed guidance for conversations about serious illness, experts at Ariadne Labs (Boston, Massachusetts, USA) developed the Serious Illness Care Program (SICP), inclusive of the Serious Illness Conversation Guide (SICG).^1^ These conversations aim to elicit seriously ill patients’ values and goals to ensure that they receive information and care that meets their needs.^2^ Studies exploring the effect of the programme and the guide have found that timely serious illness conversations can reduce patient stress and anxiety, decrease resource utilisation, result in more goal-concordant discussions and improve healthcare professionals’ experiences of care provision.^3^,^6^ While the original SICP and SICG were developed for the oncology context, in recent years both the programme and the guide have been adapted and implemented in myriad clinical settings and languages.^1 2 7^

The original SICG outlined key conversation areas, including: illness understanding, decision making and information preferences, prognostic disclosure, patient goals and fears, views on acceptable function and trade-offs and desire for family involvement^7^; however, it has been acknowledged that the guide was not comprehensive^7^ and that other important conversation domains exist.^8^ As the programme and the guide continue to be developed, adapted and implemented, it is necessary to explicate the ‘core elements’ of serious illness conversations to ensure that these components are present—or justifiably absent. For the purpose of this study, the term ‘core elements’ refers to necessary and/or important parts of serious illness conversations.^9^ The aim of this integrative systematic review was to identify and describe core elements of serious illness care conversations in the context of the SICP and/or SICG.

## Methods

### Search strategy

The search was conducted on 20 March 2023 in the bibliographic databases CINAHL, MEDLINE, PsycINFO and PubMed using the search strategy described in online supplemental material A. The search terms were established in collaboration with a university librarian. As the SICP was developed based on a literature review from 2014, the search was limited to articles published in English between 1 January 2014 and 20 March 2023. Ariadne Labs also provided a list of known publications related to the SICP (n=44).

### Eligibility criteria

Eligibility criteria were developed a priori to ensure relevance to the study aim. Articles were eligible for inclusion if they: (a) explicitly stated a connection with the SICP, SICG and/or Ariadne Labs in the title, abstract or main text and (b) provided a meaningful description of at least one serious illness conversation element. The publication language was limited to English. No restrictions were applied regarding population or setting; however, book chapters, letters to the editor and conference abstracts were excluded.

### Selection process

Known foundational articles were identified within the search results, including the original SICP development papers from Bernacki *et al*,^2 7^ which confirmed good sensitivity of the search strategy. Duplicate publications were removed. One author (SP) reviewed titles, abstracts, keywords and, when required, full-text articles against the inclusion criteria to identify eligible articles. Any uncertainty regarding initial inclusion was discussed with AS and RB. Next, full-text articles were screened for inclusion by RB and SP. Reference lists of included articles were hand searched.

### Data collection process

Three authors (RB, SP, SA) independently extracted data from six articles to calibrate the data extraction and tabulation process. Thereafter, RB extracted data by going through each article line-by-line to identify data relevant to the study aim and copying this to the extraction form described below. Only unreferenced original data were considered for extraction from the methods, results, discussion and/or conclusions sections of articles (data from the abstract, key messages, introduction and/or background sections were therefore ineligible). Unreferenced data referred to text that was presented as original without direct citation to another source. Any uncertainty regarding data eligibility was discussed between RB, SP and SA.

### Data items

An extraction form was used by RB to manually tabulate data regarding the authors, year of publication, article type, clinical context, clinicians/users, and if/how the SICP/SICG were implemented. In addition, data were extracted for tabulation regarding (a) descriptions of serious illness conversation elements and/or (b) descriptions of serious illness conversation content. This encompassed data pertaining to any time point (eg, past, present and theoretical), article type (eg, original research, case studies and clinical updates) and participant group (eg, patient, family, staff and researcher).

### Risk of bias assessment

The risk of bias was assessed using the Joanna Briggs Institute (JBI) critical appraisal checklists.^10^ These 13 checklists are used to evaluate the trustworthiness, relevance and results of published research. As there is not yet a checklist for mixed methods studies, JBI provided advice via email that the completion of more than one checklist could be appropriate for studies that enlisted more than one method. If an article presented data, even in descriptive form, one of the checklists for research studies was selected (ie, the checklist for text and opinion was not selected). Articles were assessed by responding ‘yes’, ‘no’, ‘unclear’ or ‘not applicable’ to each checklist item. If the criteria for an item were only partially fulfilled, the item was marked as ‘unclear’. One author (SP) conducted the initial critical appraisal of all articles, and any questions regarding study type or checklist selection were discussed with RB and AS. Articles were not excluded based on the appraisal responses, instead the checklists were used to inform article characteristics and comparability to support a complete discussion of the current literature. To minimise bias, JP and EKF, who authored several articles included in this review, were not involved in the article selection, data extraction or critical appraisal process.

### Synthesis methods

Thematic synthesis was selected as it provides a set of established methods for the identification of patterns and development of analytic themes in textual data.^11^,^13^ This consisted of three stages: free line-by-line coding, organisation of codes into descriptive themes and development of analytical themes.^11^ First, data were inductively interrogated for descriptions of conversation elements and coded based on the content of these descriptions. Following this, data were examined and coded for descriptions of the SICG and its content. Similar codes were compared and grouped into descriptive subthemes that remained close to the data. Lastly, the findings were synthesised and analytic themes were constructed to provide novel interpretations. The author group comprised of nurses (RB, SP, SA, AS) and physicians (EKF, JP) with experience in research and clinical practice, and extensive expertise in development and implementation of the SICP. The results were discussed and refined among the author group.

## Results

### Study selection

**Figure 1:**
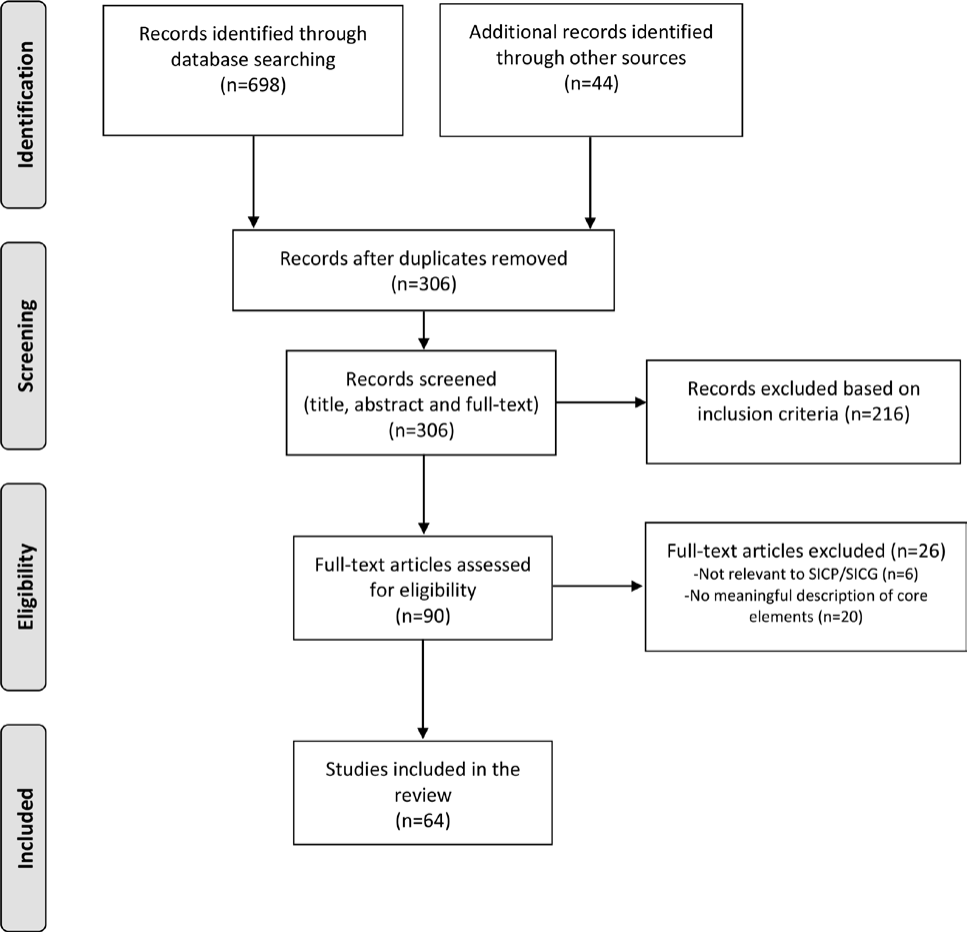
PRISMA Flow Diagram. SICG, Serious Illness Conversation Guide; SICP, Serious Illness Care Program.

The search retrieved 698 articles and a further 44 articles were provided by Ariadne Labs (figure 1). Duplicates were removed (n=436). Title, abstract and full-text screening of 306 articles were undertaken, resulting in the elimination of 216 articles. The remaining 90 full-text articles were assessed against the eligibility criteria, and the reference lists of these articles were manually searched. The reference list search revealed eight articles for full-text review; however, none met the inclusion criteria. In total, 64 articles met the inclusion criteria. Of these, 62 articles (97%) were identified through the database search and two (3%) were identified through the list provided by Ariadne Labs.

### Study characteristics

The majority of articles (n=55) were original research articles, of which 13 used qualitative methods, 15 used some form of mixed-methods and 27 used quantitative methods. Nine were categorised as text and opinion articles. Of the 64 included articles, 54 represented unique studies. Seven study clusters were identified, including the Dana-Farber Cancer Institute cluster (n=8),^45 7 14^,^18^ Brigham integrated Care Management Program cluster (n=2),^19 20^ Massachusetts General Hospital cluster 1 (n=2),^21 22^ Massachusetts General Hospital cluster 2 (n=2),^23 24^ University of Pennsylvania (n=2)^25 26^ and Meta-network Learning and Research Center Advance Care Planning cluster (n=2).^27 28^ The list of included articles, country, JBI checklist selection, clinical context and implementation/adaptation of the SICP and/or SICG is summarised in table 1.

**Table 1:** Summary of included articles, ordered by publication year

Author, year	Country	JBI checklist*	Clinical context	SICPimplementation/adaptation	SICGimplementation/adaptation
Bernacki *et al*, 2015^7^	USA	13	Oncology	Original SICP: 2.5-hour training to use SICG	Original SICG version: R4.2 12-10-13
Lakin *et al*, 2017^19^	USA	10	Primary care	Adapted SICP for the primary care setting—2.5 hours	SICG (version not stated)
Lamas *et al*, 2017^35^	USA	1	Acute care	Not applicable as interviewer was from research team	Adapted SICG for long-term acute care patients
Mandel *et al*, 2017^37^	USA	13	Nephrology	Proposed: 2.5 hours training in basic SICG competencies	Proposed: SICG (version not stated)
Miranda *et al*, 2018^14^	USA	1&9	Oncology	SICP implemented	SICG (version not stated)
O’Donnell *et al*, 2018^50^	USA	10	Heart failure	Education about ACP and role of healthcare proxy	Discussion based on SICG (version not stated)
Baran *et al*, 2019^59^	USA	13	Primary care	Not implemented	SICG version: 2015–2017
Geerse *et al*, 2019^5^	USA	9	Oncology	2.5 hours skills-based training to use the SICG	SICG version: R4.2 12-10-13
Lakin *et al*, 2019^20^	USA	9	Primary care	SIC skills training—3-hour interactive session	SICG version: R4.2 12-10-13 (2012)
Massmann *et al*, 2019^43^	USA	10	Primary care	2-hour training based on the SICP model	SICG (version not stated)
McGlinchey *et al*, 2019^63^	UK	9	UK health setting	Adapted SICP for UK healthcare setting	Adapted SICG for UK healthcare setting
Paladino *et al*, 2019^4^	USA	11	Oncology	2.5-hour skills-based training session on the SICG	SICG version: R2.7 05-25-12
Tam *et al*, 2019^52^	Canada	9&10	Internal medicine	2.5-hour small group session derived from SICP training	SICG version: 2017-04-18
Daubman *et al*, 2020^23^	USA	1	Multiple contexts	Adapted 2.5–3 hours SICG training	Modified SICG - Partners SICG
Gace *et al*, 2020^21^	USA	5	General medical	2.5 hours of SIC training modified from Ariadne Labs	SICG (version not stated)
Gelfand *et al*, 2020^40^	USA	13	Kidney care	NephroTalk, VitalTalk, Palliative Education	Adapted SICG from version: 2017-04-21
Greenwald *et al*, 2020^22^	USA	5	Medical inpatient	SICP implemented	SICG (version not stated)
Jain *et al*, 2020^29^	USA	13	Not stated	Recommends SICP training as communication resource	Refers to SICG as a communication tool
Ko *et al*, 2020^36^	Canada	1	Oncology	15 min SICG introduction, no standardised training	SICG (version not stated)
Kumar *et al*, 2020^8^	USA	1&9	Outpatient oncology	3-hour SICP structured communication education	SICG (version not stated)
Lally *et al*, 2020^53^	USA	10	Hospital patients	Communication skills training programme developed	Modified SICG for nurses (Administered by phone)
Ma *et al*, 2020^51^	Canada	10	Internal medicine	Adapted SICP, 2.5-hour workshop	SICG version: 2016
Manz *et al*, 2020^25^	USA	11	Oncology	SICG training 3 months prior to the start of the trial	ACP template based on the SICG (version not stated)
Ouchi *et al*, 2020^68^	USA	13	Emergency	Not stated	Code status conversation guide – adapted from SICG
Paladino *et al*, 2020^16^	USA	1&9	Oncology	SICP implemented	SICG version: 2015–2017
Paladino *et al*, 2020^17^	USA	11	Oncology	SICP implemented	SICG (version not stated)
Paladino *et al*, 2020^57^	USA	9&10	Health systems (3)	SICP implemented—2.5–3 hour clinician training on SIC	SICG (version not stated)
Pasricha *et al*, 2020^44^	USA	1&9	Intensive care	SICP training (3 hours)	SICG for surrogates. Based on SICG 2017-04-18
Pottash *et al*, 2020^39^	USA	1&9	Ambulatory care	Short SICG introduction, video, and role play	Adapted SICG ‘Advanced Illness Conversation Guide’
Sirianni *et al*, 2020^30^	Canada	13	COVID-19	Yes, discussed resources for SIC	SICG version: 2018-04-18
van Breemen *et al*, 2020^58^	Canada	3	Paediatrics	Training to use the SICG-Peds	Adapted SICG-Peds (12–2019)
Wasp *et al*, 2020^56^	USA	9&10	Onco & Haematology	Adapted 3-hour SICG education with 4-hour VitalTalk session	SICG Communication Skills Assessment Tool
Aaronson *et al*, 2021^38^	USA	9	Emergency	Not stated	Adapted Partners SICG for social workers
Beddard-Huber *et al*, 2021^33^	Canada	13	General	SIC interprofessional clinician workshop2.5 hour	Adapted SICG for substitute decision-makers
Daly *et al*, 2021^27^	USA	1	Family medicine	SICP implemented	SICG (version not stated)
DeCourcey *et al*, 2021^45^	USA	9	Paediatrics	Adapted PediSICP	Adapted PediSICG
Geerse *et al*, 2021^15^	USA	1&9	Oncology	2.5-hour skills-based training to use the SICG	SICG version: R4.2 12-10-13
Greenwald *et al*, 2021^41^	USA	10	Hospital setting	Clinicians participated in two 1-hour training sessions	Adapted Partners SICG—COVID-19 (03-2020)
Hafid *et al*, 2021^46^	Canada	9&10	Primary care	SICP adapted and implemented	SICG adapted (version not stated)
Karim *et al*, 2021^34^	Canada	10	Outpatient oncology	Adapted 2-hour training session based on the SICP	SICG version: 2017-04-18
Lagrotteria *et al*, 2021^54^	Canada	9	Tertiary hospitals	SICP implemented in 2.5-hour interactive training session	SICG version: draft R4.2 12-10-13
Lakin *et al*, 2021^31^	USA	10	General medicine	Adapted SICP—3-hour SAGE programme	SICG (version not stated)
Le *et al*, 2021^47^	Canada	5	Acute medicine	SIC education provided in new employee orientation	SICG (version not stated)
Moye *et al*, 2021^69^	USA	5&9	Older adults	SICP not implemented	Used six questions from the SICG
Paladino *et al*, 2021^60^	USA	9	Inpatient, outpatient	Suggests adaptation of SICP to virtual training	Adapted COVID-19 Outpatient and Inpatient Guides
Paladino *et al*, 2021^18^	USA	9	Primary care	SICP training provided	SICG version: 2017-04-18
Reed-Guy *et al*, 2021^48^	USA	1&9	Glioblastoma	SICP implemented	SICG used and adapted (version not stated)
Swiderski *et al*, 2021^71^	USA	9	Primary care	SICG training—Two 1-hour sessions	SICG (version not stated)
Thamcharoen *et al*, 2021^49^	USA	1&9	Kidney disease	Interviewer trained in SICG	Adapted SICG for researcher
Borregaard Myrhøj *et al*, 2022^32^	Denmark	9	Multiple myeloma	Team training in SIC focusing on existential issues	Modified SICG (Danish version)
Bowman *et al*, 2022^62^	USA	1	Emergency/COVID-19	SICP training provided	SICG (version not stated)
Daly *et al*, 2022^28^	USA	1	Family Medicine	Adapted SICP 1.5-hour in-person training	SICG (version not stated)
Davoudi *et al*, 2022^66^	USA	1	Oncology	SICP implemented	SICG (version not stated)
Jacobsen *et al*, 2022^24^	USA	1	Palliative care	SICP implemented	Partners SICG
Karim *et al*, 2022^42^	USA	13	Oncology	SICP training in-person or virtual workshops	SICG (version not stated)
Hu *et al*, 2022^72^	USA	1	General Surgery	Not stated	SICG (version not stated)
King *et al*, 2022^55^	Canada	1	Internal Medicine	All components of SICP implemented	SICG (version not stated)
Li *et al*, 2022^26^	USA	11	Oncology	All clinicians were trained in the use of the SICG	SICG (version not stated)
LoCastro *et al*, 2022^64^	USA	9	Haematology	Adapted SICP for delivery via telehealth	Adapted SICG for delivery via telehealth
Sanders *et al*, 2022^70^	USA	9&10	Multiple contexts	SICP 2.5-hour in-person training	SICG version: 04-2017 and revised SICG
Wasp *et al*, 2022^73^	USA	10	Oncology	3-hour SICP training in use of SICG	SICG (version not stated)
Xu *et al*, 2022^67^	USA	9	Primary Care	2.5-hour training in using SICG	SICG (version not stated)
Zehm *et al*, 2022^61^	USA	9&10	Education	Adapted SICP workshop with 2.5-hour training	Modified SICG—partners SICG
Garcia *et al*, 2023^65^	USA	9	Inpatient Clinical	Adapted team-based SICP	SICG (version not stated)

* JBI Checklist number: 1 – —analytical cross sectional studies; 2 –— case control studies; 3 – —case reports; 4 – —case Series; 5 – —cohort studies; 6 – —diagnostic test accuracy studies; 7 – —economic evaluations; 8 – —prevalence studies; 9 – —qualitative research; 10 –— quasi-experimental studies; 11 – randomized—randomised controlled trials; 12 –— systematic reviews; 13 – —text and opinion. Abbreviations: ACP – Advance Care Planning; ICU – Intensive Care Unit; – JBI; NP – Nurse practitioner; QoL – Quality of Life; SIC – Serious Illness Conversation; SICP – ; SICG – Serious Illness Conversation Program; UK – United Kingdom; USA – United States of America.

ACPadvance care planningICUintensive care unitJBIJoanna Briggs InstituteNPnurse practitionerQoLquality of lifeSICserious illness conversationSICGSerious Illness Conversation GuideSICPSerious Illness Care Program

Most articles were from North American inpatient clinical settings. Descriptions of SICP implementation and SICG version varied considerably. SICP implementation/adaptation ranged from none or unstructured training, to multiple hours of formal training. SICG implementation/adaptation was reported as including the original guide (various versions), to using a guide that had been modified for different patients, clinicians and clinical or cultural contexts.

### Critical appraisals

Most articles reported clear aims and objectives. Strategies for sampling and data collection methods were largely well defined; however, strategies for dealing with confounding factors were often not stated. In studies that reported qualitative data, there was a lack of reflection about the influence of the researcher on the research (or vice versa), and few located researchers’ cultural or theoretical backgrounds. Detailed JBI critical appraisal checklist responses are presented in online supplemental material B.

### Thematic synthesis

Three themes and six subthemes emerged to describe the core elements of serious illness conversations (see table 2).

**Table 2:** Overview of themes and subthemes

Themes	Subthemes
Serious illness conversations serve different functions that are reflected in how they are conveyed	Intentions and framingExpectations and directions
Serious illness conversations endeavour to discover what matters to patients	Current situation and possible trajectoryMatters of importance
Serious illness conversations seek to align what patients want in their life and care	Preferences and prioritiesPreparation and recommendations

### Serious illness conversations serve different functions that are reflected in how they are conveyed

The ways in which serious illness conversations were understood and conveyed impacted how the conversation was framed with respect to the clinician, the patient or the context. This theme is comprised of two subthemes: (a) intentions and framing and (b) expectations and directions.

#### Intentions and framing

The intentions and framing of the conversation described what clinicians wanted to accomplish using the guide, rather than the content of the guide itself. This included checking in, conveying medical updates or discussing the risks and benefits of treatment options,^29 30^ as well as allowing for the expression of goals, values^23 31^ and wishes and hopes for the future.^32^ Language varied when framing serious illness conversations for patients, such as: discussing future expectations,^33^ discovering what is important,^34^ conferring goals, expectations and experiences,^35^ hoping for the best and preparing for the worst,^36^ thinking and preparing,^37^ looking at the bigger picture^38^ and discussing health and future expectations.^39^

Formally introducing the conversation involved explicitly stating what it would be about, establishing an agenda, or seeking permission.^18 29 40^ Clinicians could present the conversation as an opportunity to think ahead or plan in advance^33^ in relation to the patient’s care^40^ or medical condition.^31 41^ It might be stated from the outset that the aim of the conversation was to inform future decisions and care,^33^ or the decision-making aspect could be minimised.^37 40 42^ Serious illness conversations were articulated as being part of, conceptually overlapping with, or recorded as: Advance Care Planning^57 15 24 25 27 34 36 43^,^49^ (categorised as Advance Care Planning in the electronic medical record),^4 21 23 26 31^ End-of-Life conversations,^4 7 8 14 19 31 43 47 48 50 51^ Goals-of-Care conversations^721 29 31 49 50 52^,^55^ or Values and Goals conversations (inclusive of values-based/values-centred/goals-based/goals-centred conversations).^78 17 18 20^,^23 31 33 35 42 48 56^ Conversation framing was therefore informed by diverse understandings of the concept of serious illness conversations and communicated in different ways depending on the perceived intention of the conversation.

#### Expectations and directions

Establishing expectations and directions included ascertaining what the conversation aimed to achieve, determining what subjects the conversation would address, and how much information the patient wanted or was ready to receive.^18 29 30 33^ Different versions of the SICG reflected variations in the preferred language used by clinicians, such as ‘setting up the conversation’, ‘opening the conversation’ or ‘initiating the conversation’.^33 37 40 57^ It was important to ask patients about the amount and type of information they required^30 40 49^ so that they could indicate whether they wanted (or were ready) to have an in-depth conversation about specific concerns or questions.^29 30 32 36^ By establishing expectations from the outset, the discussion could be adapted to suit the needs of the patient before providing updates or clarifications.^30^ This could help to focus only on issues that patients deemed relevant^32 36^ making the conversation less prescriptive and more collaborative.^58^

Discussing the patient’s lived experience was important to the conversation, but the ways in which this was broached varied depending on the clinician and whether the discussion was centred around the patient’s understanding of their ‘illness/medical condition’^29 30 36 40 49 57 59^ or their ‘health’.^38 41 60^ Orientating the conversation around the ‘illness’ was thought to give the clinician insight into how the patient was coping, their awareness of what was ahead and the extent to which they had accepted their illness,^29 37 59^ particularly if their function or status had changed.^58^ Centering the discussion around ‘health’ may be viewed as more holistic and could invite conversation about how patients from a variety of clinical contexts feel generally, not only in the context of their illness.^37 38 41^ It was therefore necessary to establish expectations surrounding patient understanding, acceptance, readiness and willingness early in the conversation as this could influence subsequent elements.^22 37^

### Serious illness conversations endeavour to discover what matters to patients

It was important to consider current and possible trajectories when seeking information from patients about what mattered to them in relation to their illness or health. This theme is comprised of two subthemes: (a) current situation and possible trajectory and (b) matters of importance.

#### Current situation and possible trajectory

Discussing the current situation was thought to enhance patients’ understanding of their lived reality and possible trajectories. This was termed as delivering or conveying serious news,^47^ giving medical updates,^29^ delivering prognosis,^29 61^ sharing prognosis,^44 58^ clarifying prognostic awareness^45 59 61^ and assessment of prognostic understanding.^8 16 48 51^ This subject had to be broached with care as patients could feel anxious talking (or not talking) about death or dying, and some may not want to receive prognostic information.^7 15^ Asking for permission to divulge this information was therefore an important conversation element.^29 48 52^ A prognostic discussion was still thought to be possible even if the clinician was unsure of the exact prognosis.^62^ However, prognosis might be omitted due to comfort or confidence to discuss such topics, or if it was outside clinicians’ professional scope of practice.^5 15 35 38 53^ Some elected not to focus on prognosis because discussing preferences in other domains was thought to be sufficient,^35 38^ but it was acknowledged that talking about prognosis could influence how patients answered subsequent questions.^16 37 49 58 59^ Indeed, if some form of prognosis or illness trajectory was not addressed, patients and clinicians may not be able to take full advantage of the possible benefits of the conversation.^39 40 59^

Gauging the patient’s level of trajectorial or situational awareness laid the foundation for how clinicians could clarify uncertainty for patients.^29 33 41 48^ Positive or negative wording could be used to portray information, with some recommending the use of hope/worry statements,^23 29 63^ wish/worry/wonder statements^18 33 58^ and hope for the best plan for the worst statements.^33 37^ The guide offered language templates for sharing time-based, function-based or uncertain prognoses.^7^ However, if clinicians were not comfortable providing a concrete time-based estimate, or if patients were ambivalent about receiving such information, more general information could be provided in the context of the patient’s clinical condition.^37 41 44 59 64^ This might include expected decline (ie, function, cognition, condition),^23 31 33 37 48^ expected symptoms or events related to the illness/condition,^18 46 48^ worsening trajectories,^33 47^ quality of life,^31 48^ fragility/stability^58^ and/or treatment options.^5 31^ Even if the prognosis was poor, it was important that hope and positivity was still conveyed^64^ with a view towards supporting patients through their concerns.^65^

#### Matters of importance

Discussion of important matters encompassed goals and fears, views on acceptable function (critical abilities), trade-offs and desires for family involvement.^7^ This provided opportunities for patients to express their thoughts and feelings, to discover what makes life meaningful and to reflect on important subjects to better plan care.^5 19 23 26 29 30 32 63^ The vernacular for exploring what was important varied in both the conversation guide and in the literature describing the conversation, but most encompassed some combination of the terms: values, goals, wishes, hopes, concerns, worries and fears.^45 8 20^,^23 25 27 30^ Structuring the conversation around important values and goals was viewed as differentiating serious illness conversations from other conversations in the care continuum^45^ because this focus oriented the conversation towards how the person wanted to live, not necessarily how they wanted to die.^30^ Asking about goals could lead clinicians to ask what patients would want if their goals were not within reach.^28^ The conversation could also focus on soliciting views specifically related to illness, treatment or overall care.^1822 30 33 36 38 40 44 48^,^50 56 68 69^ Value was noted in speaking about these topics more generally without necessarily linking it to an illness/health dichotomy.^29 32^ By exploring what was important through personal/clinical and concrete/existential lenses it was possible to gain insight into the patient’s experience as a person in order to construct a sensitive and appropriate way forward.^30 32 37 58 59^

Some serious illness conversations asked about sources of strength^27 33 37 40 46 47 56 57 63^ or prioritised values.^69^ Conversation elements could be added to ask about sources of support,^56^ including family support, coping resources, faith or spirituality.^45 66 70^ The subject of quality of life could likewise be introduced as a separate conversation element (eg, how would you describe your quality of life?), or it might be explored by delving deeper into the patient’s goals, worries or priorities.^32 48 49 55 56 68^ If included, questions around critical abilities explored the way that the patient wanted to live (or what they could not live without) by surveying aspects of function, purpose and meaning in the face of potentially worsening health.^16 27 33 36 40 46 48 49 53 58 63 68 69^ Eliciting the patient’s perspective regarding the functions and/or activities that were most important to them informed how to best support their needs and autonomy.^37 60 66 68^ Exploring possible trade-offs asked what patients would be willing to go through or concede in relation to, for example, gaining more time or mitigating possible losses.^27 33 36 37 40 46 49 56 58 63^ This prompted reflection and consideration of possible harms, benefits, burdens and risks related to care, as well as evaluation of what was both important and acceptable to the patient.^58^ These questions could be focused around physical or cognitive abilities^29 30^ or certain goals^48^ to give clinicians insight into acceptable care and treatments.^15 29 35 37 48^

### Serious illness conversations seek to align what patients want in their life and care

Aligning what patients wanted in their life and care involved exploring what was most significant to patients and providing appropriate recommendations and interventions based on these preferences. This theme is comprised of two subthemes: (a) preferences and priorities and (b) preparation and recommendations.

#### Preferences and priorities

It was important for patients to be able to express their preferences^4 8 17 20 42^ as this could offer clinically significant insights in relation to their overall care.^35^ However, eliciting preferences pertaining to specific medical treatments was not recommended early in the conversation as the emphasis should be on understanding the patient as a person first and foremost.^29^ Discussion of preferences and priorities might be dependent on the patient’s trajectory and whether decisions needed to be made sooner or later.^23^ This involved providing patients with dedicated time and space to ask questions, reason, deliberate and express their preferences in relation to their future.^8 32 67^

Asking about priorities was often addressed in relation to patients’ aforementioned values, goals, wishes, hopes, worries, fears and preferences.^1623 32 40 47^,^49 52 57 69^ A goal was described as a specific want or desire related to a person’s values, and a priority designated the importance of one goal or value over another.^37^ The process of prioritisation was described as asking patients what matters, and then asking them what matters most.^69^ Priorities could be explored in relation to health/illness goals and values, as well as in relation to familial, social or financial needs.^71^ Hence, it was important to ask questions to establish the meaning of a prioritised goal or value in the context of the patient’s life, and in their own words, to guide decision making and recommendations.^28 29 42 69^

#### Preparation and recommendations

Preparation of family, friends, surrogates, caregivers, healthcare proxies and substitute/medical decision makers was another important element.^40 41 49 53 56^ This included how much people in the patient’s life knew about the health/illness situation,^22^ their level of involvement^33 55 57 63^ and whether support persons had (or required) support of their own.^31^ Patients could be asked to think about who they wanted to be their substitute decision maker^36 41 47 53 60^ and prepare that person for involvement in future decision making.^30 53^ Preparation for life events such as financial planning, travel or retirement might also occur.^32 41^ Including family or caregivers in the discussion supported the identification of barriers, prompting timely action and intervention to prevent possible care or discharge delays.^72^ This was viewed as beneficial to the care partnership as it gave clinicians insight into patient and family preferences and provided family members with insight into care processes.^73^ This encouraged clinicians to not simply discuss medical events, but to consider the human character of life and illness as part of a ‘bigger picture’.^32 48^

Recommendations for ‘next steps’^28^ could be context specific^44^ or possibly dependent on the scope of practice of the clinician having the conversation.^38 53^ By eliciting aspects of life, health and illness significant to the patient, it was possible to tailor care and treatment plans that balanced the burdens/benefits of various treatment options and reflected the aspects identified as most important.^1629 31 33 40 45 58^,^60 63 67^ In this way, recommendations were not simply prescribed, but were opportunities for person-centred shared decision making.^29 30 67 68^ Discussions provided space for clinicans and patients to express their thoughts about continuing, deferring or de-escalating certain care interventions.^72^ While it was not always necessary or possible to make decisions during the conversation, it was important for patients to be prepared to make decisions with a realistic understanding of what was happening, or could happen, with their illness.^7 29 37 44 59 63^ Other care planning matters could also be addressed,^5 8 53^ including life sustaining treatments (ie, intubation, cardiopulmonary resuscitation and tracheostomy).^4 5 31 38 48 55 60 68^ Establishing code status was part of some serious illness conversations,^19 31 33 47 48 51 68 72^ but was thought to only be possible once the patient’s values and priorities were known.^4 14 30^ Others emphasised that establishing resuscitation orders should not be the focus of the conversation.^30 54^ Exploring end-of-life options could involve discussion of supportive/comfort care, hospice care, palliative care referrals and practical planning, such as assigning a healthcare proxy or establishing where the patient might like to die.^8 14 18 19 31 32 43 47 48 50 55 65 66^ Personalised preparation and recommendations provided opportunities for care to be proactive rather than reactive.^60^

## Discussion

### Main findings

This study reviewed literature pertaining to the SICP and SICG and explicated core conversation elements found therein. The three themes and six subthemes synthesise existent understandings, descriptions and interpretations of the core elements of serious illness conversations. The results revealed that the multifaceted nature and content of serious illness conversations could be framed, understood and communicated in numerous ways. While the serious illness conversation construct is relatively recent, this study has shown that its intention and subject matter is being iteratively defined and re-defined as it is adapted and applied in novel and varied contexts.

The original SICG outlined seven conversation components that were designed to support communication with patients who were often anxious due to lack of information about their prognosis or what to expect.^7^ However, this review showed that the conversation content has expanded through various adaptations to include other core elements, such as identifying a substitute or medical decision maker,^20 30 36 41 45 53^ providing clinical information,^30^ expectations for health in the future,^24 35 61^ current quality of life,^31 35 38 49^ possibility of setbacks,^35^ assess sources of strength or support,^1826 27 30 33 34 37 39 40 44^,^46 51 52 56^ end of life care,^4 14 19 31 50^ code status^14 19 30 31 33 38^ and/or other needs^14 26 41 50^; or perhaps exclude elements, such as prognosis.^35 41 53^ These changes are also seen in the fluidity of referring to serious illness conversations as advance care planning, end of life planning, goals of care conversations or values and goals conversations. Such alterations appear to reflect efforts to match differing intentions of the conversation, both from clinicians’ ‘sending’ and patients’ ‘receiving’ perspectives.

The patient and clinician-tested language of the SICG has been said to reduce the clinician’s cognitive load, while modifications to the guide align information and recommendations based on real-time feedback to match the clinical context.^31 74^ While many changes have been made to the SICG, it is worth noting that the same core elements could be used in many clinical contexts. By discussing goals, values, fears, worries, hopes, desires and wishes, in context, it becomes possible to move beyond medicalisation of the illness experience, and explore the human experience of living with a serious illness.^32^ In this way, serious illness conversations do not seek to only discuss potential life expectancy, but life expectations as a whole.^18^ These results add to the literature supporting the concept of serious illness conversations as being guided by an ethos of person-centred and goal-concordant care.

The issue of prognostication is complicated, and the various modifications made to the guide reflect that inclusion of this element may be dependent on the patient, the method of identification, the clinician’s scope of practice, and/or the clinical context.^15^ ^75^ Discussion of prognosis was viewed by some as an indicator of a high-quality serious illness conversation.^15^ This is because talking about prognosis openly could help patients with psychological and existential coping mechanisms and the day-to-day reality of living with a serious illness.^76^ Similarly, talking about what the patient experienced to be important could help them to articulate thoughts and opinions surrounding what would be acceptable to them in relation to their life and care. This highlights the importance of establishing expectations and intended outcomes for serious illness conversations in relation to the context in which they are conducted.

While the benefits (or possible drawbacks) of specific conversation domains require further exploration, this review contributes an important inventory and synthesis of existent core conversation elements. These findings pave the way for development of a conceptual framework for serious illness conversations that includes a holistic definition and content explication to further differentiate this activity in the care continuum.^76 77^ Future research could also explore the extent to which various conversation elements contribute to patient/family outcomes and clinician/organisation experiences.^8 15 76 78^

### Strengths and weaknesses

This review used rigorous methods to identify and synthesise literature pertaining to serious illness conversation core elements. Strict inclusion and exclusion criteria were adhered to, and transparent search, extraction, analysis and reporting methods were described. Thematic synthesis facilitated the analysis and inclusion of articles with varied methodologies in diverse clinical settings.

The SICP and SICG were developed by Ariadne Labs, a joint centre for health systems innovation at Brigham and Women’s Hospital and the Harvard T.H. Chan School of Public Health. As this study only examined the SICP/SICG, it is likely that these themes reflect the content of the programme and guide in some way. Other serious illness conversation training programmes or guides were not included in this study and may contain other components. Most studies originated from North America, indicating a possible lack of cultural diversity. Due to several large-scale studies and secondary analyses of data, the number of included articles outnumbers the total number of studies. However, these articles were included and analysed individually because studies originating from the same cluster explored and described different aspects of the data.

This review was not limited to study type, participant or context, and included implementation studies as well as discussion articles, so these results combine patient, clinician and researcher descriptions of serious illness conversation elements across different methods and contexts. Further, it is impossible to know how closely clinicians followed the guide, or the extent to which documentation of conversations elements in the literature reflected the actual content of conversations. The authors acknowledge their knowledge of the serious illness conversation subject area and guide content may have impacted the interpretation. The lack of a second independent initial screener of the titles and abstracts is also recognised as a limitation. Two authors in the current study authored several articles included in the current review (JP^4^,^814^ (n=16, 24.6%) and EKF^4^,^615^ (n=8, 12%)). To minimise bias, JP and EKF were not involved in article selection, data extraction or quality appraisal.

### Conclusions

This integrative systematic review explored how core elements of serious illness conversations were described in the literature and presented themes underpinning extant descriptions of these conversation elements. The results offer insights into the core elements of serious illness conversations in the context of the SICP/SICG and may be used to inform current and future clinical education and practice.

## supplementary material

10.1136/spcare-2023-004163 online supplemental file 1

## Data Availability

Data may be obtained from a third party and are not publicly available.
