# Supplementary material for: Core elements of serious illness conversations: an integrative systematic review
Source: BMJ Support Palliat Care. 2023 Jun 27;14(e3):e004163. doi: 10.1136/spcare-2023-004163 (PMC11671901; doi:10.1136/spcare-2023-004163)
Supplement: online supplemental file 1 [file spcare-14-e3-s001.pdf]

Supplemental Material A

Search strategy

Database	Search strategy	Records retrieved
**CINAHL**	AB “serious illness communication” OR AB “serious illness program*” OR AB “serious illness care” OR AB “serious illness conversation*” OR AB “serious illness model”<br>Limited to English, publications from 2014-01, academic journals.	148
**MEDLINE**	AB “serious illness communication” OR AB “serious illness program*” OR AB “serious illness care” OR AB “serious illness conversation*” OR AB “serious illness model”<br>Limited to English, publications from 2014-01, academic journals.	211
**PsychInfo**	AB “serious illness communication” OR “AB serious illness program*” OR AB “serious illness care” OR AB “serious illness conversation*” OR AB “serious illness model”<br>Limited to English, publications from 2014, academic journals.	60
**PubMed**	("serious illness communication"[Title/Abstract]) OR ((((serious illness program*[Title/Abstract]) OR (serious illness care[Title/Abstract])) OR (serious illness conversation*[Title/Abstract])) OR (serious illness model[Title/Abstract]))<br>Limited to English, publications from 2014-01-01.	279

Supplementary Material B

JBI Quality Appraisal Checklists

Checklist for Randomized Controlled Trials													
**Articles**	**Q1**	**Q2**	**Q3**	**Q4**	**Q5**	**Q6**	**Q7**	**Q8**	**Q9**	**Q10**	**Q11**	**Q12**	**Q13**
O'Donnell et al.[31]	Y	Y	U	N	N	Y	Y	Y	Y	Y	U	U	Y
Manz et al.[25]	Y	N	Y	Y	N	Y	Y	N/A	Y	Y	Y	Y	Y
Paladino et al.[17]	Y	Y	Y	Y	N	Y	Y	Y	Y	Y	U	U	Y
Paladino et al.[4]	Y	Y	Y	Y	N	Y	Y	Y	Y	Y	Y	U	Y
Li et al.[26]	Y	Y	Y	Y	U	Y	Y	N/A	Y	Y	Y	U	Y

Y = Yes; N= No; U = Unsure; N/A = Not applicable.

Tufanaru C, Munn Z, Aromataris E, Campbell J, Hopp L. Chapter 3: Systematic reviews of effectiveness. In: Aromataris E, Munn Z (Editors). JBI Manual for Evidence Synthesis. JBI, 2020. Available from https://synthesismanual.jbi.global

Checklist for Quasi-Experimental Studies									
**Articles**	**Q1**	**Q2**	**Q3**	**Q4**	**Q5**	**Q6**	**Q7**	**Q8**	**Q9**
Lakin et al.[55]	Y	N	Y	Y	N	Y	Y	Y	Y
Karim et al.[53]	Y	U	U	N	N	N/A	Y	Y	Y
Wasp et al.*[47]	U	Y	U	N	N	N/A	Y	U	U
Ma et al.[40]	Y	Y	Y	N	N	N/A	Y	Y	U
Paladino et al.*[42]	N	Y	N	N	N	N/A	Y	U	Y
Massman et al.[33]	U	Y	U	N	N	N/A	Y	U	U
Tam et al.*[35]	Y	Y	Y	N	N	N/A	Y	U	Y
Lally et al.[39]	N	U	Y	N	N	N/A	Y	U	U
Lakin et al.[19]	U	Y	U	Y	N	N/A	Y	Y	U
Zehm et al.*[72]	U	Y	U	N	N	N/A	Y	U	U
Hafid et al.*[52]	U	Y	Y	N	N	N/A	Y	U	U
Sanders et al.*[69]	U	Y	U	N	N	N/a	Y	U	U
Wasp et al.[70]	N	U	U	N	N	N/A	Y	U	U

Y = Yes; N= No; U = Unclear; N/A = Not applicable; * = mixed method/multiple checklists.

Tufanaru C, Munn Z, Aromataris E, Campbell J, Hopp L. Chapter 3: Systematic reviews of effectiveness. In: Aromataris E, Munn Z (Editors). JBI Manual for Evidence Synthesis. JBI, 2020. Available from https://synthesismanual.jbi.global

Checklist for Cohort Studies											
**Articles**	**Q1**	**Q2**	**Q3**	**Q4**	**Q5**	**Q6**	**Q7**	**Q8**	**Q9**	**Q10**	**Q11**
Le et al.[56]	Y	Y	Y	Y	U	U	Y	Y	U	Y	Y
Greenwald et al.[22]	Y	Y	U	Y	U	U	U	Y	Y	Y	Y
Gace et al.[21]	U	U	U	Y	U	U	Y	U	N	N	Y

Y = Yes; N= No; U = Unclear; N/A = Not applicable.

Moola S, Munn Z, Tufanaru C, Aromataris E, Sears K, Sfetcu R, Currie M, Qureshi R, Mattis P, Lisy K, Mu P-F. Chapter 7: Systematic reviews of etiology and risk . In: Aromataris E, Munn Z (Editors). JBI Manual for Evidence Synthesis. JBI, 2020. Available from https://synthesismanual.jbi.global

Checklist for Analytical Cross Sectional Studies								
**Articles**	**Q1**	**Q2**	**Q3**	**Q4**	**Q5**	**Q6**	**Q7**	**Q8**
Thamcharoen et al.*[61]	Y	Y	Y	Y	U	U	U	Y
Greenwald et al.[51]	Y	Y	U	U	N	N	U	Y
Kumar et al.*[8]	Y	Y	Y	Y	Y	N	U	Y
Geerse et al.*[15]	Y	Y	U	U	Y	U	U	Y
Paladino et al.*[16]	Y	Y	U	U	Y	N	U	Y
Miranda et al.*[14]	Y	Y	U	Y	Y	N	U	Y
Lamas et al.[29]	Y	Y	Y	Y	N	N	U	Y
Daubman et al.[23]	Y	Y	U	U	N	N	U	U

Ko et al.[38]	N	N	Y	U	Y	Y	U	Y
Pasricha et al.*[43]	Y	Y	U	Y	Y	N	U	Y
Daly et al.[27]	Y	Y	U	U	Y	U	U	Y
Jacobsen et al.[24]	N	Y	U	U	U	N	U	Y
Reed-Guy et al.*[59]	Y	Y	U	Y	Y	N	U	Y
Moye et al.*[57]	Y	Y	U	Y	Y	Y	U	Y
Pottash et al.*[44]	Y	U	U	Y	N	N	U	Y
Bowman et al.[63]	Y	Y	U	U	Y	N	U	Y
Daly et al.[28]	Y	Y	U	U	N	N	U	Y
Davoudi et al.[64]	Y	Y	U	U	Y	U	U	Y
Hu et al.[66]	Y	Y	U	U	U	N	U	Y
King et al.[67]	Y	Y	Y	Y	Y	Y	U	Y

Y = Yes; N= No; U = Unclear; N/A = Not applicable; * = mixed method/multiple checklists.

Moola S, Munn Z, Tufanaru C, Aromataris E, Sears K, Sfetcu R, Currie M, Qureshi R, Mattis P, Lisy K, Mu P-F. Chapter 7: Systematic reviews of etiology and risk . In: Aromataris E, Munn Z (Editors). JBI Manual for Evidence Synthesis. *JBI, 2020. Available from https://synthesismanual.jbi.global*

**Checklist for Qualitative Research**										
**Articles**	**Q1**	**Q2**	**Q3**	**Q4**	**Q5**	**Q6**	**Q7**	**Q8**	**Q9**	**Q10**
Thamcharoen et al *[61]	Y	Y	Y	Y	Y	Y	U	Y	Y	Y
Paladino et al.[58]	Y	Y	U	U	U	Y	N	U	N	U
DeCourcey et al.[50]	Y	Y	Y	Y	Y	Y	Y	Y	N	Y
Paladino et al.[18]	Y	Y	Y	Y	Y	Y	U	Y	Y	Y
Kumar et al.*[8]	Y	Y	Y	Y	Y	N	U	Y	Y	Y
Geerse et al.*[15]	U	Y	Y	U	U	N	U	U	Y	Y
Wasp et al.*[47]	U	U	Y	Y	Y	N	N	Y	Y	U
Paladino et al.*[16]	Y	Y	Y	Y	Y	N	U	Y	Y	Y
Paladino et al.*[42]	Y	Y	Y	Y	Y	N	U	Y	Y	Y
Tam et al.*[35]	U	U	Y	U	Y	N	N	Y	Y	Y
McGlinchey et al.[34]	Y	Y	Y	Y	Y	N	N	Y	Y	Y
Geerse et al.[5]	Y	Y	Y	Y	Y	Y	Y	Y	Y	Y
Lakin et al.[20]	Y	Y	Y	Y	Y	Y	Y	Y	Y	Y
Miranda et al.*[14]	U	U	U	U	U	N	N	Y	Y	U
Pasricha et al.*[43]	U	Y	U	U	U	N	U	Y	Y	Y
Zehm et al.*[72]	U	U	Y	U	U	N	N	U	N	Y
Borregaard Myrhøj et al.[62]	Y	Y	Y	Y	Y	Y	Y	Y	N	Y
Reed-Guy et al.*[59]	Y	Y	Y	U	Y	N	N	Y	Y	Y
Lagrotteria et al.[54]	Y	Y	Y	Y	Y	Y	Y	Y	Y	Y
Hafid et al.*[52]	Y	Y	Y	U	U	Y	N	U	Y	Y
Swiderski et al.[60]	Y	Y	Y	Y	Y	Y	Y	Y	N	Y
Aaronson et al.[48]	Y	Y	Y	Y	Y	N	N	N	Y	Y
Moye et al.*[57]	Y	Y	Y	Y	Y	Y	Y	Y	Y	Y
Pottash et al.*[44]	U	Y	Y	U	U	N	N	U	Y	Y
LoCastro et al.[68]	U	Y	Y	Y	Y	N	U	Y	Y	Y
Sanders et al.*[69]	U	Y	Y	Y	Y	U	U	Y	Y	Y
Xu et al.[71]	U	Y	Y	Y	Y	N	U	Y	Y	Y
Garcia et al.[73]	Y	Y	Y	Y	Y	Y	U	Y	Y	Y

Y = Yes; N= No; U = Unclear; N/A = Not applicable; * = mixed method/multiple checklists.

Lockwood C, Munn Z, Porritt K. Qualitative research synthesis: methodological guidance for systematic reviewers utilizing meta-aggregation. Int J Evid Based Healthc. 2015;13(3):179–187.

**Checklist for Case Reports**								
**Articles**	**Q1**	**Q2**	**Q3**	**Q4**	**Q5**	**Q6**	**Q7**	**Q8**
Van Breemen et al.[46]	Y	Y	Y	Y	Y	Y	Y	Y

Y = Yes; N= No; U = Unclear; N/A = Not applicable.

Moola S, Munn Z, Tufanaru C, Aromataris E, Sears K, Sfetcu R, Currie M, Qureshi R, Mattis P, Lisy K, Mu P-F. Chapter 7: Systematic reviews of etiology and risk. In: Aromataris E, Munn Z (Editors). JBI Manual for Evidence Synthesis. JBI, 2020. Available from https://synthesismanual.jbi.global

Checklist for Text and Opinion						
**Articles**	**Q1**	**Q2**	**Q3**	**Q4**	**Q5**	**Q6**
Beddard-Huber et al.[49]	Y	Y	Y	Y	Y	Y
Gelfand et al.[36]	Y	Y	Y	Y	Y	Y
Sirianni et al.[45]	Y	Y	Y	Y	Y	Y
Jain et al.[37]	Y	Y	Y	Y	Y	Y
Baran et al.[32]	Y	Y	Y	Y	Y	Y
Mandel et al.[30]	Y	Y	Y	Y	Y	Y
Bernacki et al.[7]	Y	Y	Y	Y	Y	Y
Ouchi et al.[41]	Y	Y	Y	Y	Y	Y
Karim et al.[65]	Y	Y	Y	Y	Y	Y

Y = Yes; N= No; U = Unsure; N/A = Not applicable.

McArthur A, Klugarova J, Yan H, Florescu S. Innovations in the systematic review of text and opinion. Int J Evid Based Healthc. 2015;13(3):188–195.
